# Supplementary material for: Learning curve and surgical outcome of robotic assisted colorectal surgery with ERAS program
Source: Sci Rep. 2022 Nov 29;12:20566. doi: 10.1038/s41598-022-24665-w (PMC9709162; doi:10.1038/s41598-022-24665-w)
Supplement: Supplementary file 2 — Supplementary Information 2. [file 41598_2022_24665_MOESM2_ESM.zip › RAL Raw data-Ñ[▒K/048/HS_ñw╝╨░O▒Kñσ.pdf]

正式摘要(電子簽章：00000206626180)

W52 / W52 ☐ 外文標題(注意:無英文姓名-請補病人基本檔!) ☐【讀取】憑證

臺中榮民總醫院

出院病歷摘要

轉入號： 轉出號：

[redacted]

(8)流水編號	
(9)入院日期	109/12/20 (2020) 直腸外科 W52 - 145
(10)轉科日期	
(11)出院日期	109/12/31 (2020) 住院天數計 11 日

診斷

- 入院 :1.rectal cancer, 4cm FAV, s/p CCRT
  2.type 2 diabete mellitus
- 出院 :1.rectal cancer, 4cm FAV, s/p CCRT, s/p robot assisted low anterior resection and loop ileostomy on 2020/12/25
  2.type 2 diabete mellitus

主訴

- bloody stool since this August

病史

- This 55 years old female patient has past history of type 2 diabete mellitus and she recieved hysterectomy 20 years ago. She complained bloody stool and tenesmus since this August. Decreased body weight was also noted. She recieved colonoscopy and ructum tumor was found 4cm from anal verge and biopsy showed malignancy. Abdominal CT showed tumor sized about 6.6cm with perirectal lymphnode metastasis. She recieved CCRT from 9/8 to 10/20. Follow-up CT showed regressive changed. Thus, she was admitted for pre-operation survey.

體檢發現

-
  - General appearance :
    - easy looking , conscious clear , without cardiopulmonary distress
    - Vital signs自動帶入date 體溫(BT) 脈搏(P) 呼吸(R) 血壓(BP) 身高(BH) 體重(BW) BMI
      20201220 36℃ 94次/分 18次/分 111/77mmHg 150.8cm 50.9kg 22.4
  - HEENT :
    - Eyes: no anemic conjunctiva, no icteric sclera, normal EOM
    - ENT: no otorrhea, no rhinorrhea, no erythematous mucosa, no injected tonsil, no deformity
  - Neck: supple, freely movable, trachea midline,no deformity, no engorged jugular vein , no palpable thyroid or lymph node enlargement
  - Chest:
    - Inspection: Grossly normal, symmetric and fully expansion, no accessory muscle used, no operation scar, no spider angiomata, no gynecomastia
    - Palpation: no palpable mass or nodule, no subcutaneous emphysema, no tenderness
    - Ausculation: clear breathing sound
  - Heart:
    - Percussion: normal heart size
    - Ausculation: regular heart beat, normal S1, S2, no gallop, no murmur
  - Abdomen:
    - Inspection: in
      - flat contour, symetrically, no scar, no stria, no dilated veins, no rashes
    - Ausculation: normoactive bowel sound, no abdominal bruits
    - palpation: soft, no muscle guarding or rigidity, no local tenderness, no rebounding pain, no palpable mass, no palpable pulsation
    - Percussion: no shifting dulllness, no hepatomegaly, no splenomagaly
  -

Peripheral pulsation :	CA	BA	RA	FA	PA	PTA	DPA
Right side :	++	++	++	++	++	++	++/+++
Left side :	++	++	++	++	++	++	++/+++

  - Genito-anus: no tenderness or palpable mass lesion or rectal shelf, normal external genitalia, normal anal tone, brown stool

CRS CONDITION

-
  - Lung

手術日期及方法

- 手術日期及方法20201226 Robot Assisted Low Anterior Resection

住院治療經過

- After admission, we arranged colonoscopy that showed regressive change of previous rectal tumor. Operation with robot assisted low anterior resection with loop ileostomy was done on 12/25. Under ERAS protocol, we let her try water and then diet and stop IVF on POD2. Ostomy rod was removed on 12/30. Under the stable condition, she was discharged on 12/31.

合併症

- nil

一般檢查

- 累積報告 :

DATE	TIME	ALB	TP	BIL.T	BIL.D	ALKP	AST	ALT	LDH	CHOL	TG	HDL-C	LDL	UA	NH3	AMY
1091224	0926	4.4		0.90				11								
1091226	1722	4.0		0.63	0.18	80	17	11								
1091231	0900	3.4		0.63				15								

DATE	TIME	WBC	RBC	HGB	HCT	MCV	PLT	BLST	NEUT	BAND	SEG	LYM	MON	EOS	BAS
1091224	0902	3220	4.64	13.8			153		60.9			14.3	9.9	14.0	0.9
1091226	1721	9100	4.63	13.6	41.3	89.2	138		95.0			2.5	2.3	0.1	0.1
1091231	0855	4990		12.5	37.8		142		73.0			7.6	7.8	11.2	0.4

DATE	TIME	NA	K	CL	CA	BUN	CREAT	Mg	P	CRP	ESR	PCT	LIPASE	LACTATE
1091224	0926	142	3.9	102	9.2	19	0.58							
1091226	1722	142	3.2	107	8.2	9	0.50							
1091231	0900	142	4.1			10	0.59							

DATE	TIME	GLUCOSE	PAB	TRF	ZN	CU	KETONE	CK	CKMB	PT-P	PT-C	APTT-P	APTT-C	TRO-T
1091226	1722	165												

- 放射線報告 :檢驗項目 **:MRI - PELVIS** 報告日期 **:1091229**
  主 訴：
  Result:
  Finding:
  MRI of pelvis was performed and the images revealed:
  > History of rectal cancer, S/P biopsy proof and neoadjuvant CCRT.
  > Presence of wall thickening appearance over rectum, about 68 mm in long axis, with perirectal infiltrative appearance. Mild decrease in focal wall thickness is noted as compared with previous CT study on 20200820.
  The previously mentioned regional metastatic lymphadenopathy(s) could not be certainly identified as suspicious lymphadenopathy in this MRI study.
  Rectal cancer with perirectal invasion and regional lymph node metastasis S/P CCRT with post-Tx changes could be compatible.
  Please correlate with clinical condition.

- > Focal prominence over bilateral adnexal areas. Please correlate with clinical condition.
  > Suspect small amount of ascites or focal fluid accumulation over pelvic area.
  > Relatively small size of the urinary bladder with mild wall thickening appearance. Please correlate with clinical condition.
  > Non-visualization of uterus, and S/P OP appearance over pelvis. Please correlate with clinical condition.
  Impression:
  Please see descriptions.

特殊檢查

病理報告

其它

出院時情況

- 3.改門診治療

出院指示

藥名	劑量	劑量單位	用法	頻次	數量	數量單位	現況	天數	單位
500mg Glucophage	1	TAB	PO	BID	14	TAB	TH	7	天
Ultracet tab	1	TAB	PO	QIDPRN	28	TAB	TH	7	天

出院計劃

主治醫師 2062F 蔣鋒帆 / 住院醫師 2331F 陳凱祥

正式摘要(電子簽章：000000206626180)

W52 / W52

doctorRank:B
